# Dimensional Structure and Cultural Invariance of DSM V Post-traumatic Stress Disorder Among Iraqi and Syrian Displaced People

**DOI:** 10.3389/fpsyg.2019.01505

**Published:** 2019-07-09

**Authors:** Hawkar Ibrahim, Claudia Catani, Azad Ali Ismail, Frank Neuner

**Affiliations:** ^1^Department of Psychology, Clinical Psychology and Psychotherapy, Bielefeld University, Bielefeld, Germany; ^2^Department of Clinical Psychology, Koya University, Koya, Iraq; ^3^Vivo International, Konstanz, Germany

**Keywords:** PTSD, DSM, confirmatory factor analysis, measurement invariance, Kurd, Arab, Iraqi, Syrian

## Abstract

While the factor structure of post-traumatic stress disorder (PTSD) symptoms has been investigated among various traumatized populations in Western and high-income countries, knowledge regarding the validity of factor structure of PTSD among culturally diverse populations in low-and-middle-income countries is limited. The current study examined the factor structure and cultural invariance of PTSD in 521 Iraqi and 993 Syrian war-affected displaced people who were living in the Kurdistan Region of Iraq. Results from confirmatory factor analyses demonstrated that alternative factor models for PTSD, including a new model derived from this population (anhedonia and affect model) resulted in a better fit than the current DSM V models. Taken together, the results showed that a good fit, as well as the measurement invariance of PTSD factors, could be obtained by applying the anhedonia and hybrid model. This study provides further support for the anhedonia and hybrid model of PTSD and fills an important gap in knowledge about the validity of PTSD symptom clusters among Arab and Kurdish populations.

## Introduction

Post-traumatic stress disorder (PTSD) is a common psychological disorder worldwide (Atwoli et al., 2015). According to the World Health Organization's (WHO) mental health surveys, estimates of lifetime prevalence rates range between 3.9 and 5.6% (Koenen et al., 2017). While these numbers document the burden of PTSD, the estimated frequency of PTSD among refugees and war-affected populations in low- and middle-income countries (LMIC) is many times higher than in populations that live in peace (Fazel et al., 2005; Steel et al., 2009). Since the concept of PTSD and related assessment instruments were developed and tested mainly in peaceful high-income countries, from a global perspective, it is of the upmost importance to test the validity of the criteria among culturally diverse, heavily affected populations.

Prominent examples of war-affected societies include the Middle East regions affected by the wars in Syria and Iraq. Since the Arab Spring movement brought social and political instability to this region in 2011, this area has been considered as one of the top global hotspots for insurgencies and war. The ongoing civil wars in Syria and Iraq have resulted in one of the largest migration waves in modern history. According to the United Nations High Commissioner for Refugees (UNHCR), from the start of the Arab Spring events in early 2011 until the end of 2017, more than three million Iraqi people and nearly half of Syria's population have been displaced (United Nations High Commissioner for Refugees, 2018). Studies have shown that, due to what they had experienced during the war and flight, displaced people are more likely to develop psychological problems (Scholte et al., 2004; Husain et al., 2011). Although there is a lack of solid studies in the Middle East, the research available among small to medium samples suggests that the Iraqi and Syrian displaced people had been exposed to an extraordinarily wide range of war-related events, with high rates of PTSD (Quosh et al., 2013; Taylor et al., 2014; Hassan et al., 2016; Ibrahim and Hassan, 2017; Kazour et al., 2017; AlShawi, 2018).

Post-traumatic stress disorder is a psychological disorder characterized by a set of symptom clusters that could occur following the witnessing or experiencing of a traumatic event. Although it has been a matter of some debate since its introduction into the Diagnostic and Statistical Manual of Mental Disorders (DSM-III; American Psychiatric Association, 1980) in 1980, the factor structure of PTSD symptoms has remained remarkably robust for decades. The DSM-III classified PTSD as an anxiety disorder with its symptoms characterized by re-experiencing the traumatic event (B criteria), numbing of responsiveness to or reduced involvement with the external world (C criteria), and variety of autonomic, dysphoric, or cognitive symptoms (D criteria; American Psychiatric Association, 1980). The DSM-III was revised in 1987 (American Psychiatric Association, 1987), and PTSD symptoms were expanded, but the clusters remained the same. In the DSM-IV (American Psychiatric Association, 2000) some notable differences were introduced relating to the definition of the traumatic event types that were required as gate-criterion for a diagnosis of PTSD, but left the three symptom clusters unaffected.

The first major changes of symptom clusters were introduced in the current version of the DSM (DSM-V; American Psychiatric Association, 2013). The most significant modification was dividing three symptom clusters into four clusters; intrusion (criterion B), avoidance (criterion C), negative alterations in cognition and mood (criterion D), and trauma-related alterations in arousal and reactivity (criterion E). According to the fifth edition of the DSM, in order to make a diagnosis of PTSD, the person must have been exposed to a direct or indirect traumatic event, such as indirect exposure; witnessing the trauma, learning that a relative or close friend was exposed to a trauma, or other forms of indirect exposure, such as exposure to trauma during the professional duties (criterion A). In addition, the person must have at least one symptom of B and C criterion with at least two symptoms of D and E criterion.

However, since the introduction of PTSD as a disorder, empirical tests of the defined PTSD clusters have found only limited validity of the criteria. On the basis of factor analyses, several alternative models for PTSD symptom clusters have been proposed. King et al. (1998) tested the factor structure of PTSD assessed with the gold-standard clinician-administered PTSD scale (CAPS) among treatment-seeking male military veterans. They proposed a new latent model for PTSD, the emotional numbing model, and found that this model provides the best fit to data in contrast to the DSM-IV model. While the DSM-IV gathered the avoidance and numbing symptoms under the single category of avoidance, the emotional numbing model separated avoidance symptoms from numbing symptoms and put them into two independent factors. The resulting emotional numbing model differentiated the four symptom clusters re-experiencing, effortful avoidance, emotional numbing, and hyperarousal. The emotional numbing model remained the most prominent alternative model for the DSM IV diagnosis of PTSD until Simms et al. (2002) suggested the dysphoria model. The dysphoria model consists of the four factors: re-experiencing, avoidance, dysphoria, and hyperarousal. In this model, the re-experiencing and avoidance factors were identical with the DSM-IV, but two symptoms represented the hyperarousal symptom cluster (hypervigilance and exaggerated startle response), and the remaining symptoms were gathered under the dysphoria factor. Simms et al. (2002) examined their model among a large sample of deployed Gulf War veterans (*N* = 3,695) and documented that the dysphoria model provided the best fit to the data. Elhai et al. (2011) combined both four-factor models and suggested the dysphoric arousal model. This model consists of five factors; re-experiencing, avoidance, numbing, dysphoric arousal, and anxious arousal.

After the introduction of the DSM V, the dysphoric arousal model inspired other researchers to propose and test several alternative models for PTSD symptom clusters. In the first large-scale factor analysis that was carried out outside of the United States, Liu et al. (2014a) examined the dimensions of DSM V PTSD symptoms among Chinese earthquake survivors and proposed new model for PTSD, the anhedonia model, that consisted of six symptom clusters—intrusion, avoidance, negative affect, anhedonia, dysphoric arousal, and anxious arousal. In their study Liu et al. (2014a) found that the anhedonia model fits the data significantly better than other competing models. At the same time, Tsai et al. (2015) extended the dysphoric arousal model and anhedonia model by suggesting the externalizing behaviors model by introducing a new factor (externalizing behavior) that consisted of two PTSD symptoms, irritability or aggression and risky or destructive behavior. More recently, the seven-factor hybrid model has been proposed by Armour et al. (2015) as a result of combining two of the aforementioned six-factor models (anhedonia and externalizing behavior).

To date, the four-factor model of the DSM V criteria for PTSD has been studied among different traumatized populations, including survivors of natural disasters (Liu et al., 2014a; Wang et al., 2015a; Mordeno and Hall, 2017), veterans (Hall et al., 2012; Keane et al., 2014; Armour et al., 2015; Tsai et al., 2015; Bovin et al., 2016; Konecky et al., 2016), military service members (Wortmann et al., 2016), refugees (Schnyder et al., 2015; Specker et al., 2018) and other traumatized people (Hafstad et al., 2014; Armour et al., 2015; Ashbaugh et al., 2016; Yang et al., 2017). In general, these studies found that the four-factor DSM V model showed a sufficient fit with their data, but performed inferior to alternative PTSD models, especially the anhedonia and hybrid models. However, according to our best knowledge, no large-scale study has been carried out in a war-affected population, the cultural variance of the populations studied to date is limited and does not include any Middle East sample. Therefore, in the current study, we aimed to examine the factor structure of PTSD symptoms among Iraqi and Syrian displaced people who are living in the Kurdistan Region of Iraq (KRI). In particular, we aimed to test the fit of the DSM V model in comparison to the main alternative models. This study builds upon a previous translation of the PTSD Checklist for DSM-5 (PCL-5) into Arabic and Kurdish and its validation in this population (Ibrahim et al., 2018a). All participants in the current study share a common feature in that they fled from their homes due to war and persecution. However, the sample was heterogeneous regarding three factors: nationality, language, and ethnicity. The current study also aimed to compare the cultural invariance of the PTSD factor structure using the DSM V and alternative models for PTSD. The examination of factor structure and cultural invariance of PTSD symptom clusters among non-Western and low-income populations is important as it expands the cross-cultural understanding of PTSD structure and it will provide significant information about the impact of language, ethnicity, and culture on the validity of PTSD symptom clusters.

## Materials and Methods

### Participants and Procedure

The study sample was drawn from a collaborative research project between Bielefeld University in Germany and Koya University in the KRI. A survey was carried out between December 2016 and July 2017, where 26 locally trained clinical psychologists and social workers interviewed unselected 521 Iraqi IDPs and 993 Syrian refugees. Using standardized written informed consent sheet, participants were fully informed about the procedures of the study. Due to cultural considerations (Ibrahim and Hassan, 2017), verbal informed consent was given. The study and its procedure, including the reliance on verbally informed consents, were approved by the Ethical Committee of Bielefeld University in Germany as well as the Ethical Committee of Koya University in the KRI.

Due to the absence of reliable census data from the displaced camps, a random selection of individuals and households was not possible, therefore we used a pragmatic sampling approach based on a random selection of individuals and households. The camps were subdivided into 6–7 sections by the camp administrations, and tents were chosen based on a random selection of households by spinning a pen from the zone center. Trained interviewers visited the household that was in a straight line from the tip of the pen, identified the people in the household and determined their eligibility for participation in the study. The details of ethical considerations, as well as the data collection procedure and sample selection, are described elsewhere (Ibrahim et al., 2018a,b).

The majority of the participants were female (63%). About one third (31%) of the participants were illiterate, and 87.1% of them had no regular monthly income, others had between 30,000 and 1,200,000 IDQ (1,000 IDQ ≈ 0.65 Euro local rate). Due to the variety of official languages in the northern regions of Syria and Iraq, participants were free to choose between either Kurdish and Arabic languages during their interview. As a result, 55.5% of the full sample (12.3% of Iraqis and 78.2% of Syrian) were interviewed in Kurdish and the rest were interviewed in Arabic (see Table 1).

**Table 1:** Sociodemographic information.

**Mean age (SD)^a^**	**33.73 (11.49)**
**Gender**, ***N*** **(%)**	
Male	548 (36.2)
Female	966 (63.8)
**Religion**, ***N*** **(%)**	
Muslim	1,060 (70)
Yazidi	454 (30)
**Ethnicity**, ***N*** **(%)**	
Kurd	1,160 (76.6)
Arab	354 (23.4)
**Nationality**, ***N*** **(%)**	
Iraqi	521 (34.4)
Syrian	993 (65.6)
**Formal education, mean (SD)**^**a**^^,^ ^b^	5.83 (25.92)
**Occupation**, ***N*** **(%)**	
Currently working	407 (26.9)
Currently non-working	1,107 (73.1)
**Language of interview**	
Kurdish	841 (55.5)
Arabic	673 (44.5)
**Having regular income**, ***N*** **(%)**	
No	1,304 (86.1)
Yes	210 (13.9)
**Individual monthly income, mean (SD)**^c^^,^ ^d^	17,045.90 (91,753.87)
**Number of children, mean (SD)**^**e**^	3.49 (2.76)
Number of boys^f^	1.80 (1.65)
Number of girls^f^	1.68 (1.67)

a *In year*.

b *Score range: 0–20*.

c *In Iraqi Dinar*.

d *Score range:0–1,200,000 IQD*.

e *Score range: 0–14*.

f *Score range: 0–9*.

### Instruments

#### PTSD Checklist for DSM-5 (PCL-5)

Kurdish and Arabic translations of the PCL-5 that had been validated in the cultural context previously (Ibrahim et al., 2018a) were used to assess PTSD symptoms. The PCL-5 is a self-report scale that records the presence and severity of each DSM-V PTSD symptom. The severity and frequency of each of the 20 DSM-V symptoms on the PCL-5 are rated from 0 (not at all) to 4 (extremely); the total sum score ranges from 0 to 80, higher scores indicate a greater number of and more intense PTSD symptoms. With the PCL-5, the PTSD diagnosis can be obtained by two different methods. First, using a DSM-V diagnostic algorithm for PTSD, treating each DSM-V symptom criterion as fulfilled if the corresponding PCL-5 item that was rated as ≥2 (at least “moderately”), then following the DSM-5 diagnostic rule for PTSD (which is required at least one symptom from of B and C criterion and at least two symptoms of D and E criterion). Second, by using suggested cutoff values; a total score of ≥33 and ≥23 has been suggested by Weathers et al. (2013b) and Ibrahim et al. (2018a), respectively, for a probable diagnosis of PTSD. The internal consistency of the PCL-5 was high (Cronbach's α = 0.92).

#### Depression Scale of the Hopkins Symptom Checklist-25 (DHSCL-25)

Kurdish and Arabic versions of the DHSCL-25 (Ibrahim et al., 2018a) were used to examine the intensity and presence of symptoms of depression. The DHSCL-25 is a self-report inventory that consists of 15 items that are rated on a four-point scale from 1 (not at all) to 4 (extremely). The DHSCL-25 demonstrated a good internal consistency (Cronbach's α = 0.88).

#### War and Adversity Exposure Checklist (WAEC)

Based on focus group discussions with displaced Iraqi and Syrian people and including items from different sources [e.g., War Exposure Scale (Ibrahim et al., 2018a) and Life Events Checklist for the DSM-5 (Weathers et al., 2013a)] we have developed the WAEC (Ibrahim et al., 2018b). It consists of 25 items with “Yes” and “No” answers that evaluate general and war-related traumatic event exposure. The Cronbach's alpha for internal consistency of the WAEC was 0.88.

### Data Analysis

The normal distribution of the PCL-5 sum-score, as well as the subscale scores in each single factor in all different models were checked by examining the indicators of skewness and kurtosis along with a visual inspection of the corresponding histograms, normal Q–Q plots, and boxplots. Results showed that all scales were normally distributed. To investigate the underlying dimensions of the PCL-5, an exploratory factor analysis was performed using a maximum likelihood approach with an oblique rotation method. The number of factors was determined based on eigenvalues >1 and scree plot.

To verify the factor structure of each PTSD model, a set of confirmatory factor analyses (CFA) was carried out. Model fit was evaluated on the basis of various fit indices. Since the chi-square test is extremely sensitive to sample size, this test was considered inadequate for the large sample size of this study (Bentler and Bonett, 1980). Therefore, we relied on other fit indices including Comparative Fit Index (CFI), Tucker-Lewis index (TLI), Root-Mean-Square Error of Approximation (RMSEA), and Standardized Root-Mean-Square Residual (SRMR). Cut-off values for each fit index were drawn from standard publications (for review see: Bentler, 1992; Hu and Bentler, 1999; Cheung and Rensvold, 2002; Schermelleh-Engel et al., 2003; Kline, 2011). A model was considered to be acceptable if the values of CFI and TLI were ≥0.90, RMSEA ≤ 0.06, and criteria for the RMSEA 90% confidence interval were met [lower value <0.05, and upper value <0.08] as well as SRMR <0.08. Since non-nested models do not allow for the test of Chi-square values, we counted on differences in Bayesian Information Criterion (BIC) values. As suggested by Raftery (1995) lower BIC values indicate a better model fit, and lower values with 10-point difference yield very strong support for a better fit.

The measurement invariance was investigated by testing the invariance of the presumed underlying constructs of the PCL-5 across the sample subgroups, divided by nationality (Iraqi and Syrian), language of the interview (Kurdish and Arabic), and ethnicity (Kurd and Arab). For this purpose, sequential steps of the CFA with single-groups as well as across groups were conducted (Steenkamp and Baumgartner, 1998; Chen et al., 2005; van de Schoot et al., 2012). First, to test the validity of configural invariance in each single group a set of CFAs were conducted. Second, when single group CFA was confirmed, we carried out the same CFA across groups (as also referred to as multigroup confirmatory factor analysis (MCFA) to test configural invariance by constraining factor structures to be equal among groups. Third, when configural invariance across groups was supported, metric invariance was tested by constraining factor loadings to be equal across groups. Last, after achieving metric invariance, we constrained the intercepts of items to be similar across groups in order to examine scalar invariance.

All descriptive statistics were carried out using the Statistical Package for Social Sciences (SPSS-Windows version 25). The relationship between continuous variables was examined using Pearson's correlation coefficient. A *t*-test was utilized to identify mean differences between groups. The confirmatory factor analysis and measurement invariance was analyzed using Amos 25.

## Results

### Trauma Characteristics and Probable PTSD Diagnosis

Results from descriptive statistics showed that participants had been exposed to between 0 and 20 traumatic event types in their lifetime (*M* = 6.43, *SD* = 3.69). Between the two nationality groups participating in the study, 98.8% (*M* = 6.71, *SD* = 3.91) of Iraqis and 98.5% (*M* = 6.29, *SD* = 3.56) of Syrian participants had experienced at least one traumatic event in their lifetime. The mean sum score of the PCL-5 was significantly higher among Iraqis when compared to Syrian participants (*M* = 44.66 vs. *M* = 26.52, *p* = 0.000). Using the DSM-V diagnostic algorithm for PTSD 43% of all participants (64.1% Iraqis and 31.9% Syrian) met the full criteria for probable PTSD diagnosis. In addition, using the contextual adapted cutoff value of 23 (Ibrahim et al., 2018a), more than two-thirds (67.6%) of participants had a sum score of 23 or higher, and 50% of them had a score equal to or above the 33 cutoff value suggested by Weathers et al. (2013b).

### Data-Dependent Model Resulting From Exploratory Factor Analysis: Anhedonia and Affect Model

A maximum likelihood exploratory factor analysis with oblique rotation method was conducted first. Results from a Kaiser-Meyer-Olkin (KMO) test of sampling adequacy and Bartlett's test of sphericity showed that the data meets the factorability criteria (KMO = 0.938 and Bartlett's test [$\chi_{\left(190\right)}^{2}$ = 13,224.36, *p* = 0.000]. Using the scree plot and eigenvalue >1.0 criterion, results indicated four factors which accounted for 49.45% of the variance. Intrusion items loaded on the first factor. Negative alterations in cognitions items loaded in two separate factors (factors 2 and 3) that could be interpreted as “anhedonia” and “negative changes in beliefs and affect.” Avoidance items highly loaded in factor 4 (the item factor loadings are presented in Table 2).

**Table 2:** Factor loading matrix of PCL-5 for each factor.

**#**	**Items**	**Factor loading**			
		**In**	**NCBA**	**AN**	**AV**
2	Repeated, disturbing dreams of the stressful experience?	0.794	0.140	−0.148	−0.120
1	Repeated, disturbing, and unwanted memories of the stressful experience?	0.753	−0.139	0.071	0.045
4	Feeling very upset when something reminded you of the stressful experience?	0.749	−0.173	0.111	0.079
5	Having strong physical reactions when something reminded you of the stressful experience?	0.667	0.032	0.071	0.035
3	Suddenly feeling or acting as if the stressful experience were actually happening again?	0.479	0.181	0.059	0.056
11	Having strong negative feelings, such as fear, horror, anger, guilt, or shame?	0.365	0.170	0.148	0.030
16	Taking too many risks or doing things that could cause you harm?	−0.196	0.676	0.110	0.008
17	Being “super alert” or watchful or on guard?	0.163	0.611	−0.111	0.060
18	Feeling jumpy or easily startled?	0.380	0.549	−0.162	−0.056
8	Trouble remembering important parts of the stressful experience?	−0.051	0.480	−0.030	0.123
19	Having difficulty concentrating?	0.026	0.480	0.200	−0.024
20	Trouble falling or staying asleep?	0.140	0.458	0.138	−0.057
10	Blaming yourself or someone else for the stressful experience or what happened after it?	0.021	0.432	0.134	0.054
15	Irritable behavior, angry outbursts, or acting aggressively?	0.189	0.403	0.158	−0.083
9	Having strong negative beliefs about yourself, other people, or the world?	−0.042	0.362	0.251	0.104
14	Trouble experiencing positive feelings?	0.051	0.017	0.757	−0.021
12	Loss of interest in activities that you used to enjoy?	0.157	−0.026	0.657	0.006
13	Feeling distant or cut off from other people?	−0.075	0.168	0.568	−0.051
6	Avoiding memories, thoughts, or feelings related to the stressful experience?	0.037	0.031	−0.032	0.882
7	Avoiding external reminders of the stressful experience?	−0.013	0.054	−0.015	0.880

*In, intrusion symptoms; NCBA, negative changes in beliefs and affect; AN, anhedonia; AV, avoidance*.

As shown in Table 3, to keep a minimum consistency with all other PTSD models that show a high agreement in the intrusion factor, such as the DSM-V model (American Psychiatric Association, 2013), dysphoria (Simms et al., 2002), dysphoric arousal (Elhai et al., 2011), anhedonia (Liu et al., 2014a), externalizing behaviors (Tsai et al., 2015), and Hybrid model (Armour et al., 2015), we moved item 11 from the intrusion factor to the negative changes in beliefs and affect factor and defined this new model as the “anhedonia and affect model” (see Table 3 for a comparison with other models). All factors of this empirically derived model had an acceptable internal consistency (see Table 4) Moreover, all factors were significantly and meaningfully correlated with each other and with the sum scores of traumatic events, PTSD, and depression (see Table 5).

**Table 3:** Item distribution of PTSD symptoms based on seven PTSD models.

**Symptoms**	**DSM5 model**	**Anhedonia and affect**	**Dysphoria**	**Dysphoric arousal**	**Externalizing behaviors**	**Anhedonia**	**Hybrid**
1. Repeated, disturbing, and unwanted memories of the stressful experience?	IN	IN	IN	IN	IN	IN	IN
2. Repeated, disturbing dreams of the stressful experience?	IN	IN	IN	IN	IN	IN	IN
3. Suddenly feeling or acting as if the stressful experience were actually happening again?	IN	IN	IN	IN	IN	IN	IN
4. Feeling very upset when something reminded you of the stressful experience?	IN	IN	IN	IN	IN	IN	IN
5. Having strong physical reactions when something reminded you of the stressful experience?	IN	IN	IN	IN	IN	IN	IN
6. Avoiding memories, thoughts, or feelings related to the stressful experience?	AV	AV	AV	AV	AV	AV	AV
7. Avoiding external reminders of the stressful experience?	AV	AV	AV	AV	AV	AV	AV
8. Trouble remembering important parts of the stressful experience?	NACM	NCBA	DY	NACM	NACM	NA	NA
9. Having strong negative beliefs about yourself, other people, or the world?	NACM	NCBA	DY	NACM	NACM	NA	NA
10. Blaming yourself or someone else for the stressful experience or what happened after it?	NACM	NCBA	DY	NACM	NACM	NA	NA
11. Having strong negative feelings, such as fear, horror, anger, guilt, or shame?	NACM	NCBA	DY	NACM	NACM	NA	NA
12. Loss of interest in activities that you used to enjoy?	NACM	AN	DY	NACM	NACM	AN	AN
13. Feeling distant or cut off from other people?	NACM	AN	DY	NACM	NACM	AN	AN
14. Trouble experiencing positive feelings?	NACM	AN	DY	NACM	NACM	AN	AN
15. Irritable behavior, angry outbursts, or acting aggressively?	AAR	NCBA	DY	DYA	EB	DYA	EB
16. Taking too many risks or doing things that could cause you harm?	AAR	NCBA	AAR	DYA	EB	DYA	EB
17. Being “super alert” or watchful or on guard?	AAR	NCBA	AAR	AA	AA	AA	AA
18. Feeling jumpy or easily startled?	AAR	NCBA	AAR	AA	AA	AA	AA
19. Having difficulty concentrating?	AAR	NCBA	DY	DYA	DYA	DYA	DYA
20. Trouble falling or staying asleep?	AAR	NCBA	DY	DYA	DYA	DYA	DYA

*IN, intrusion symptoms; AV, avoidance; NACM, negative alterations in cognitions and mood; AAR, alterations in arousal and reactivity; AN, anhedonia; NCBA, negative changes in beliefs and affect; DY, dysphoria; DYA, dysphoric arousal; AA, anxious arousal; EB, externalizing behaviors; NA, negative affect*.

**Table 4:** Reliability estimates of the PTSD dimensions in different models.

**Models**		**Total sample**		**Nationality**				**Language of interview**				**Ethnicity**			
		**Cronbach's α**	**95.0% CI**	**Iraqi**		**Syrian**		**Kurdish**		**Arabic**		**Kurd**		**Arab**	
				**Cronbach's α**	**95.0% CI**	**Cronbach's α**	**95.0% CI**	**Cronbach's α**	**95.0% CI**	**Cronbach's α**	**95.0% CI**	**Cronbach's α**	**95.0% CI**	**Cronbach's α**	**95.0% CI**
DSM 5	IN	0.848	[0.835, 0.859]	0.809	[0.782, 0.834]	0.810	[0.790, 0.828]	0.814	[0.793, 0.833]	0.841	[0.821, 0.859]	0.828	[0.811, 0.843]	0.841	[0.821, 0.859]
	AV	0.892	[0.880, 0.902]	0.896	[0.877, 0.913]	0.881	[0.865, 0.895]	0.858	[0.838, 0.876]	0.923	[0.910, 0.934]	0.888	[0.875, 0.901]	0.923	[0.910, 0.934]
	NACM	0.797	[0.781, 0.812]	0.784	[0.755, 0.811]	0.761	[0.738, 0.783]	0.777	[0.753, 0.799]	0.785	[0.759, 0.809]	0.787	[0.768, 0.806]	0.785	[0.759, 0.809]
	AAR	0.813	[0.799, 0.828]	0.784	[0.754, 0.812]	0.742	[0.716, 0.766]	0.756	[0.729, 0.780]	0.809	[0.789, 0.831]	0.782	[0.762, 0.801]	0.809	[0.786, 0.831]
Anhedonia and affect	IN	0.848	[0.835, 0.859]	0.809	[0.782, 0.834]	0.810	[0.790, 0.828]	0.814	[0.793, 0.833]	0.841	[0.821, 0.859]	0.828	[0.811, 0.843]	0.841	[0.821, 0.859]
	AV	0.892	[0.880, 0.902]	0.896	[0.877, 0.913]	0.881	[0.865, 0.895]	0.858	[0.838, 0.876]	0.923	[0.910, 0.934]	0.888	[0.875, 0.901]	0.923	[0.910, 0.934]
	NCBA	0.854	[0.843, 0.865]	0.816	[0.791, 0.839]	0.760	[0.737, 0.782]	0.804	[0.784, 0.823]	0.853	[0.836, 0.869]	0.828	[0.813, 0.843]	0.825	[0.796, 0.851]
	AN	0.762	[0.740, 0.782]	0.725	[0.681, 0.763]	0.769	[0.742, 0.792]	0.786	[0.759, 0.810]	0.714	[0.674, 0.749]	0.764	[0.740, 0.787]	0.724	[0.670, 0.771]
Dysphoria	IN	0.848	[0.835, 0.859]	0.809	[0.782, 0.834]	0.810	[0.790, 0.828]	0.814	[0.793, 0.833]	0.841	[0.821, 0.859]	0.828	[0.811, 0.843]	0.841	[0.821, 0.859]
	AV	0.892	[0.880, 0.902]	0.896	[0.877, 0.913]	0.881	[0.865, 0.895]	0.858	[0.838, 0.876]	0.923	[0.910, 0.934]	0.888	[0.875, 0.901]	0.923	[0.910, 0.934]
	DY	0.862	[0.851, 0.872]	0.849	[0.829, 0.868]	0.830	[0.814, 0.845]	0.838	[0.822, 0.854]	0.857	[0.840, 0.872]	0.847	[0.833, 0.860]	0.844	[0.818, 0.867]
	AAR	0.733	[0.704, 0.758]	0.666	[0.603, 0.718]	0.623	[0.573, 0.667]	0.663	[0.579, 0.679]	0.729	[0.685, 0.767]	0.712	[0.677, 0.743]	0.613	[0.524, 0.686]
Dysphoric arousal	IN	0.848	[0.835, 0.859]	0.809	[0.782, 0.834]	0.810	[0.790, 0.828]	0.814	[0.793, 0.833]	0.841	[0.821, 0.859]	0.828	[0.811, 0.843]	0.841	[0.821, 0.859]
	AV	0.892	[0.880, 0.902]	0.896	[0.877, 0.913]	0.881	[0.865, 0.895]	0.858	[0.838, 0.876]	0.923	[0.910, 0.934]	0.888	[0.875, 0.901]	0.923	[0.910, 0.934]
	NACM	0.797	[0.781, 0.812]	0.784	[0.755, 0.811]	0.761	[0.738, 0.783]	0.777	[0.753, 0.799]	0.785	[0.759, 0.809]	0.787	[0.768, 0.806]	0.785	[0.759, 0.809]
	DYA	0.735	[0.713, 0.756]	0.715	[0.673, 0.753]	0.669	[0.634, 0.701]	0.686	[0.649, 0.719]	0.730	[0.695, 0.762]	0.701	[0.672, 0.728]	0.714	[0.662, 0.760]
	AA	0.733	[0.704, 0.758]	0.666	[0.603, 0.718]	0.623	[0.573, 0.667]	0.663	[0.579, 0.679]	0.729	[0.685, 0.767]	0.712	[0.677, 0.743]	0.613	[0.524, 0.686]
Externalizing behaviors	IN	0.848	[0.835, 0.859]	0.809	[0.782, 0.834]	0.810	[0.790, 0.828]	0.814	[0.793, 0.833]	0.841	[0.821, 0.859]	0.828	[0.811, 0.843]	0.841	[0.821, 0.859]
	AV	0.892	[0.880, 0.902]	0.896	[0.877, 0.913]	0.881	[0.865, 0.895]	0.858	[0.838, 0.876]	0.923	[0.910, 0.934]	0.888	[0.875, 0.901]	0.923	[0.910, 0.934]
	NCBA	0.854	[0.843, 0.865]	0.816	[0.791, 0.839]	0.760	[0.737, 0.782]	0.804	[0.784, 0.823]	0.853	[0.836, 0.869]	0.828	[0.813, 0.843]	0.825	[0.796, 0.851]
	EB	0.578	[0.534, 0.619]	0.559	[0.476, 0.629]	0.495	[0.428, 0.554]	0.535	[0.468, 0.594]	0.556	[0.484, 0.619]	0.540	[0.484, 0.590]	0.553	[0.449, 0.637]
	AA	0.733	[0.704, 0.758]	0.666	[0.603, 0.718]	0.623	[0.573, 0.667]	0.663	[0.579, 0.679]	0.729	[0.685, 0.767]	0.712	[0.677, 0.743]	0.613	[0.524, 0.686]
	DYA	0.647	[0.609, 0.681]	0.624	[0.553, 0.683]	0.588	[0.533, 0.636]	0.589	[0.530, 0.641]	0.656	[0.600, 0.705]	0.618	[0.571, 0.659]	0.609	[0.519, 0.683]
Anhedonia	IN	0.848	[0.835, 0.859]	0.809	[0.782, 0.834]	0.810	[0.790, 0.828]	0.814	[0.793, 0.833]	0.841	[0.821, 0.859]	0.828	[0.811, 0.843]	0.841	[0.821, 0.859]
	AV	0.892	[0.880, 0.902]	0.896	[0.877, 0.913]	0.881	[0.865, 0.895]	0.858	[0.838, 0.876]	0.923	[0.910, 0.934]	0.888	[0.875, 0.901]	0.923	[0.910, 0.934]
	NA	0.659	[0.631, 0.687]	0.662	[0.612, 0.707]	0.528	[0.479, 0.574]	0.573	[0.523, 0.618]	0.671	[0.621, 0.709]	0.604	[0.565, 0.640]	0.652	[0.589, 0.708]
	AN	0.762	[0.740, 0.782]	0.725	[0.681, 0.763]	0.769	[0.742, 0.792]	0.786	[0.759, 0.810]	0.714	[0.674, 0.749]	0.764	[0.740, 0.787]	0.724	[0.670, 0.771]
	DYA	0.647	[0.609, 0.681]	0.624	[0.553, 0.683]	0.588	[0.533, 0.636]	0.589	[0.530, 0.641]	0.656	[0.600, 0.705]	0.618	[0.571, 0.659]	0.609	[0.519, 0.683]
	AA	0.733	[0.704, 0.758]	0.666	[0.603, 0.718]	0.623	[0.573, 0.667]	0.663	[0.579, 0.679]	0.729	[0.685, 0.767]	0.712	[0.677, 0.743]	0.613	[0.524, 0.686]
Hybrid	IN	0.848	[0.835, 0.859]	0.809	[0.782, 0.834]	0.810	[0.790, 0.828]	0.814	[0.793, 0.833]	0.841	[0.821, 0.859]	0.828	[0.811, 0.843]	0.841	[0.821, 0.859]
	AV	0.892	[0.880, 0.902]	0.896	[0.877, 0.913]	0.881	[0.865, 0.895]	0.858	[0.838, 0.876]	0.923	[0.910, 0.934]	0.888	[0.875, 0.901]	0.923	[0.910, 0.934]
	NA	0.659	[0.631, 0.687]	0.662	[0.612, 0.707]	0.528	[0.479, 0.574]	0.573	[0.523, 0.618]	0.671	[0.621, 0.709]	0.604	[0.565, 0.640]	0.652	[0.589, 0.708]
	AN	0.762	[0.740, 0.782]	0.725	[0.681, 0.763]	0.769	[0.742, 0.792]	0.786	[0.759, 0.810]	0.714	[0.674, 0.749]	0.764	[0.740, 0.787]	0.724	[0.670, 0.771]
	EB	0.578	[0.534, 0.619]	0.559	[0.476, 0.629]	0.495	[0.428, 0.554]	0.535	[0.468, 0.594]	0.556	[0.484, 0.619]	0.540	[0.484, 0.590]	0.553	[0.449, 0.637]
	AA	0.733	[0.704, 0.758]	0.666	[0.603, 0.718]	0.623	[0.573, 0.667]	0.663	[0.579, 0.679]	0.729	[0.685, 0.767]	0.712	[0.677, 0.743]	0.613	[0.524, 0.686]
	DYA	0.647	[0.609, 0.681]	0.624	[0.553, 0.683]	0.588	[0.533, 0.636]	0.589	[0.530, 0.641]	0.656	[0.600, 0.705]	0.618	[0.571, 0.659]	0.609	[0.519, 0.683]

*IN, intrusion symptoms; AV, avoidance; NACM, negative alterations in cognitions and mood; AAR, alterations in arousal and reactivity; AN, anhedonia; NCBA, negative changes in beliefs and affect; DY, dysphoria; DYA, dysphoric arousal; AA, anxious arousal; EB, externalizing behaviors; NA, negative affect*.

**Table 5:** Correlation between PTSD factors with depression and number of experienced traumatic events.

**PTSD factors**	**Trauma sum score**		**Depression sum score**	
	**Semi partial correlations**	**Zero-order correlations**	**Semi partial correlations**	**Zero-order correlations**
**HYBRID MODEL**				
IN	0.054^*^	0.260^**^	0.059^**^	0.602^**^
AV	0.059^*^	0.196^**^	0.001	0.347^**^
NA	0.046	0.258^**^	0.072^**^	0.608^**^
AN	0.061^*^	0.248^**^	0.173^**^	0.653^**^
DYA	0.005	0.214^**^	0.262^**^	0.709^**^
EB	0.064^*^	0.242^**^	0.132^**^	0.611^**^
AA	0.007	0.218^**^	0.002	0.532^**^
**ANHEDONIA MODEL**				
IN	0.054^*^	0.260^**^	0.059^**^	0.602^**^
AV	0.059^*^	0.196^**^	0.006	0.347^**^
NA	0.052^*^	0.258^**^	0.081^**^	0.608^**^
AN	0.049^*^	0.248^**^	0.100^**^	0.653^**^
DYA	0.080^**^	0.244^**^	0.531^**^	0.870^**^
AA	0.012	0.218^**^	0.025^*^	0.532^**^
**ANHEDONIA AND AFFECT MODEL**				
IN	0.051^*^	0.260^**^	0.046^*^	0.602^**^
NCBA	0.088^**^	0.286^**^	0.324^**^	0.746^**^
AN	0.063^*^	0.248^**^	0.200^**^	0.653^**^
AV	0.058^*^	0.196^**^	−0.018	0.347^**^

*NOTE: IN, intrusion symptoms; AV, avoidance; AN, anhedonia; NA, negative affect; NCBA, negative changes in beliefs and affect; DYA, dysphoric arousal; AA, anxious arousal; EB, externalizing behaviors*.

* *p < 0.05*.

** *p < 0.01*.

### Factor Structure of PTSD

As presented in Table 6, a multigroup CFA based on each PTSD model was undertaken. Results showed that each different PTSD model met acceptable fit criteria (all CFIs ≥ 0.92, TLIs ≥ 0.91, RMSEAs ≤ 0.06, and SRMRs <0.04). In a non-nested comparison, the anhedonia model demonstrated a better fit than the hybrid (Δ BIC of 16.689) and the anhedonia and affect models (Δ BIC of 124.861).

**Table 6:** Multi group confirmatory factor analysis based on PTSD models.

**Models**		**Goodness of fit indexes**					
	**χ^2^ (df)**	**CFI**	**TLI**	**RMSEA**	**RMSEA 90% CI**	**SRMR**	**BIC**
DSM-5	1,104.924 (164)^**^	0.928	0.917	0.062	[0.058, 0.065]	0.041	1,441.760
Anhedonia and affect	995.775 (164)^**^	0.940	0.930	0.056	[0.053, 0.060]	0.037	1,292.611
Dysphoria	1,141.818 (164)^**^	0.925	0.914	0.063	[0.059, 0.066]	0.042	1,478.653
Dysphoric arousal	980.514 (160)^**^	0.937	0.926	0.058	[0.055, 0.062]	0.039	1,346.639
Externalizing behaviors	958.753 (155)^**^	0.939	0.925	0.059	[0.055, 0.062]	0.039	1,361.491
Anhedonia	765.012 (155)^**^	0.953	0.943	0.051	[0.047, 0.055]	0.033	1,167.750
Hybrid	737.766 (149)^**^	0.955	0.943	0.051	[0.047, 0.055]	0.032	1,184.439

** *p = 0.000*.

### Measurement Invariance

Single group CFAs (nationality—Iraqi and Syrian; the language of interview—Kurdish and Arabic; ethnicity—Kurd and Arab) for each PTSD model were conducted and results showed that the anhedonia and hybrid models were good at all relevant criteria (CFIs ≥ 0.90, TLIs ≥ 0.90, RMSEAs ≤ 0.06, and SRMRs <0.04; see Table 7). With single group CFAs of these two models was supported, in the next step, factor structures in all two models were constrained to be equal across groups to test configural invariance. As presented in Table 8, all models provided a good fit of configural invariance to the data. Metric invariance was examined by constraining all factor loadings to be equal across groups and results showed that both models achieved an acceptable level of all fit indices. In the final step, scalar invariance through constraining the intercepts of items to be equal across groups was assessed and results demonstrated that the scalar invariance of the anhedonia and hybrid models yielded a good fit across groups (Table 8 reports goodness of fit indices for measurement invariance). Standardized estimates for factor loadings, based anhedonia and hybrid models for every single group was shown in Table 9.

**Table 7:** Single group confirmatory factor analysis based on PTSD models.

**Models**	**Goodness of fit indexes**					
	**χ^2^ (df)**	**CFI**	**TLI**	**RMSEA**	**RMSEA 90% CI**	**SRMR**
**DSM-5**						
Iraqi	525.29 (164)^**^	0.91	0.89	0.06	[0.059, 0.071]	0.04
Syrian	843.06 (164)^**^	0.90	0.88	0.06	[0.060, 0.069]	0.04
Kurdish	777.76 (164)^**^	0.89	0.88	0.06	[0.062, 0.072]	0.04
Arabic	595.18 (164)^**^	0.92	0.91	0.06	[0.057, 0.068]	0.04
Kurd	923.50 (164)^**^	0.91	0.90	0.06	[0.059, 0.067]	0.04
Arab	428.25 (164)^**^	0.90	0.88	0.06	[0.060, 0.075]	0.05
**ANHEDONIA AND AFFECT**						
Iraqi	533.64 (164)^**^	0.90	0.89	0.06	[0.060, 0.072]	0.04
Syrian	761.11 (164) ^**^	0.91	0.90	0.06	[0.056, 0.065]	0.04
Kurdish	700.69 (164)^**^	0.89	0.90	0.06	[0.058, 0.067]	0.04
Arabic	570.01 (164)^**^	0.93	0.91	0.06	[0.055, 0.066]	0.04
Kurd	808.10 (164)^**^	0.92	0.91	0.05	[0.054, 0.062]	0.04
Arab	440.94 (164)^**^	0.89	0.87	0.06	[0.061, 0.077]	0.05
**DYSPHORIA**						
Iraqi	552.22 (164)^**^	0.90	0.88	0.05	[0.061, 0.074]	0.06
Syrian	863.37 (164)^**^	0.89	0.88	0.06	[0.061, 0.070]	0.04
Kurdish	800.39 (164)^**^	0.89	0.87	0.06	[0.063, 0.073]	0.05
Arabic	460.01 (164)^**^	0.88	0.87	0.07	[0.058, 0.069]	0.05
Kurd	943.16 (164)^**^	0.91	0.90	0.06	[0.060, 0.068]	0.04
Arab	460.01 (164)^**^	0.88	0.87	0.07	[0.064, 0.079]	0.05
**DYSPHORIC AROUSAL**						
Iraqi	479.48 (160)^**^	0.92	0.91	0.06	[0.056, 0.068]	0.04
Syrian	782.43 (160) ^**^	0.91	0.89	0.06	[0.058, 0.067]	0.04
Kurdish	731.50 (160)^**^	0.90	0.88	0.06	[0.060, 0.070]	0.04
Arabic	526.53 (160)^**^	0.93	0.92	0.05	[0.053, 0.064]	0.04
Kurd	816.24 (160)^**^	0.92	0.91	0.05	[0.055, 0.064]	0.04
Arab	408.05 (160)^**^	0.90	0.88	0.06	[0.058, 0.074]	0.05
**EXTERNALIZING BEHAVIORS**						
Iraqi	740.88 (155)^**^	0.92	0.90	0.06	[0.056, 0.069]	0.04
Syrian	757.12 (155) ^**^	0.91	0.89	0.06	[0.058, 0.067]	0.04
Kurdish	711.02 (155)^**^	0.90	0.88	0.06	[0.061, 0.070]	0.04
Arabic	516.19 (155)^**^	0.93	0.92	0.05	[0.053, 0.065]	0.04
Kurd	790.06 (155)^**^	0.92	0.91	0.05	[0.055, 0.064]	0.04
Arab	399.77 (155)^**^	0.90	0.88	0.06	[0.059, 0.075]	0.05
**ANHEDONIA**						
Iraqi	424.73 (155)^**^	0.93	0.91	0.05	[0.051, 0.064]	0.04
Syrian	663.32 (155)^**^	0.92	0.91	0.05	[0.053, 0.062]	0.04
Kurdish	606.13 (155)^**^	0.92	0.90	0.05	[0.054, 0.064]	0.04
Arabic	460.45 (155)^**^	0.94	0.93	0.05	[0.048, 0.060]	0.03
Kurd	663.29 (155)^**^	0.94	0.93	0.05	[0.049, 0.057]	0.03
Arab	349.20 (155)^**^	0.92	0.91	0.06	[0.051, 0.068]	0.04
**HYBRID**						
Iraqi	416.00 (149)^**^	0.93	0.91	0.05	[0.052, 0.065]	0.04
Syrian	638.85 (149)^**^	0.93	0.91	0.05	[0.052, 0.062]	0.04
Kurdish	584.01 (149)^**^	0.92	0.90	0.05	[0.054, 0.064]	0.04
Arabic	447.56 (149)^**^	0.94	0.93	0.05	[0.049, 0.060]	0.03
Kurd	627.07 (149) ^**^	0.94	0.93	0.05	[0.048, 0.057]	0.03
Arab	339.81 (149)^**^	0.92	0.90	0.06	[0.052, 0.069]	0.04

** *p = 0.000*.

**Table 8:** Testing measurement invariance across groups based on anhedonia and hybrid models.

**Anhedonia model**	**Goodness of fit indexes**					
**Cross groups**	**χ^2^ (df)**	**CFI**	**TLI**	**RMSEA**	**RMSEA 90% CI**	**SRMR**
**NATIONALITY**						
Configural invariance	1,088.110 (310)^**^	0.92	0.91	0.04	[0.038, 0.043]	0.04
Metric invariance	1,206.252 (324)^**^	0.91	0.90	0.04	[0.040, 0.045]	0.05
Scalar invariance	1,331.118 (345)^**^	0.91	0.90	0.04	[0.041, 0.046]	0.08
**LANGUAGE OF INTERVIEW**						
Configural invariance	1,066.579 (310)^**^	0.93	0.92	0.04	[0.038, 0.043]	0.04
Metric invariance	1,164.512 (324)^**^	0.92	0.91	0.04	[0.039, 0.044]	0.04
Scalar invariance	1,276.287 (345)^**^	0.92	0.91	0.04	[0.040, 0.045]	0.06
**ETHNICITY**						
Configural invariance	1,012.723 (310)^**^	0.940	0.926	0.039	[0.036, 0.041]	0.03
Metric invariance	1,061.266 (324)^**^	0.937	0.926	0.039	[0.036, 0.041]	0.03
Scalar invariance	1,156.890 (345)^**^	0.930	0.923	0.039	[0.037, 0.042]	0.03
**Hybrid model**	**Goodness of fit indexes**					
**Cross groups**	**χ^2^ (df)**	**CFI**	**TLI**	**RMSEA**	**RMSEA 90% CI**	**SRMR**
**NATIONALITY**						
Configural invariance	1,044.91 (298)^**^	0.93	0.91	0.04	[0.038, 0.043]	0.04
Metric invariance	1,161.84 (311)^**^	0.92	0.90	0.04	[0.040, 0.045]	0.05
Scalar invariance	1,295.11 (339)^**^	0.91	0.90	0.04	[0.041, 0.046]	0.08
**LANGUAGE OF INTERVIEW**						
Configural invariance	1,031.57 (298)^**^	0.93	0.92	0.04	[0.038, 0.043]	0.04
Metric invariance	1,130.79 (311)^**^	0.93	0.91	0.04	[0.039, 0.044]	0.04
Scalar invariance	1,244.73 (339)^**^	0.92	0.91	0.04	[0.040, 0.045]	0.06
**ETHNICITY**						
Configural invariance	967.118 (298)	0.94	0.92	0.03	[0.036, 0.041]	0.03
Metric invariance	1,012.247 (311)	0.94	0.92	0.03	[0.036, 0.041]	0.03
Scalar invariance	1,123.460 (339)	0.93	0.92	0.03	[0.037, 0.042]	0.03

** *p = 0.000*.

**Table 9:** Standardized estimates for factor loadings, based anhedonia and hybrid models for each single group.

**Items**	**Anhedonia model**						**Hybrid model**					
	**Nationality**		**Language of interview**		**Ethnicity**		**Nationality**		**Language of interview**		**Ethnicity**	
	**Iraqi**	**Syrian**	**Kurdish**	**Arabic**	**Kurd**	**Arab**	**Iraqi**	**Syrian**	**Kurdish**	**Arabic**	**Kurd**	**Arab**
1	0.66	0.66	0.67	0.69	0.68	0.64	0.66	0.66	0.67	0.69	0.68	0.64
2	0.70	0.61	0.60	0.75	0.66	0.70	0.70	0.60	0.60	0.75	0.66	0.70
3	0.63	0.67	0.67	0.68	0.69	0.66	0.63	0.67	0.67	0.68	0.69	0.66
4	0.68	0.72	0.72	0.70	0.71	0.64	0.68	0.72	0.72	0.70	0.71	0.64
5	0.75	0.71	0.72	0.76	0.74	0.72	0.75	0.71	0.72	0.76	0.74	0.73
6	0.88	0.92	0.89	0.92	0.90	0.86	0.88	0.92	0.90	0.92	0.90	0.86
7	0.91	0.85	0.83	0.92	0.88	0.91	0.91	0.85	0.83	0.92	0.88	0.91
8	0.36	0.27	0.30	0.45	0.36	0.35	0.36	0.20	0.30	0.44	0.35	0.36
9	0.68	0.47	0.48	0.65	0.54	0.66	0.68	0.46	0.47	0.65	0.54	0.67
10	0.68	0.47	0.52	0.63	0.54	0.65	0.68	0.48	0.52	0.63	0.54	0.65
11	0.62	0.60	0.62	0.62	0.63	0.63	0.62	0.60	0.62	0.63	0.63	0.63
12	0.72	0.76	0.78	0.71	0.76	0.69	0.72	0.76	0.78	0.71	0.77	0.69
13	0.61	0.62	0.63	0.58	0.61	0.63	0.61	0.62	0.63	0.58	0.61	0.62
14	0.72	0.81	0.83	0.72	0.80	0.72	0.72	0.81	0.83	0.72	0.79	0.72
15	0.61	0.63	0.66	0.62	0.63	0.66	0.65	0.72	0.73	0.65	0.69	0.68
16	0.56	0.50	0.54	0.56	0.54	0.55	0.59	0.54	0.58	0.59	0.59	0.56
17	0.64	0.71	0.72	0.70	0.74	0.62	0.64	0.70	0.71	0.70	0.74	0.62
18	0.77	0.63	0.64	0.81	0.73	0.71	0.77	0.64	0.64	0.81	0.74	0.70
19	0.67	0.58	0.59	0.68	0.61	0.64	0.69	0.61	0.63	0.70	0.69	0.66
20	0.62	0.64	0.63	0.67	0.66	0.61	0.65	0.67	0.66	0.69	0.64	0.65

### Additional Analyses

As shown in the Table 10, the psychogenic amnesia symptom (item 8) had low factor loadings across all models. Therefore, we conducted CFAs without item 8 based on all models. Results showed that all models were improved by the removal of item 8 (see Tables 11, 12). Further, following previously presented results, the anhedonia model remained a better-fitted model when compared to all models, including a hybrid model, with (Δ BIC of 12.907).

**Table 10:** Standardized estimates for factor loadings.

**Items**	**DSM V**	**Anhedonia and affect**	**Dysphoria**	**Dysphoric arousal**	**Externalizing behaviors**	**Anhedonia**	**Hybrid**
1	0.709	0.709	0.709	0.709	0.709	0.709	0.709
2	0.722	0.723	0.723	0.723	0.723	0.723	0.723
3	0.716	0.716	0.716	0.716	0.716	0.716	0.716
4	0.721	0.721	0.721	0.721	0.721	0.721	0.721
5	0.765	0.765	0.765	0.764	0.765	0.764	0.765
6	0.910	0.911	0.911	0.910	0.910	0.906	0.906
7	0.885	0.884	0.883	0.885	0.884	0.889	0.888
8	0.427	0.445	0.429	0.426	0.425	0.450	0.447
9	0.588	0.571	0.576	0.590	0.589	0.595	0.594
10	0.579	0.577	0.569	0.577	0.577	0.594	0.595
11	0.635	0.636	0.634	0.629	0.630	0.644	0.646
12	0.710	0.774	0.688	0.709	0.709	0.769	0.769
13	0.564	0.610	0.556	0.570	0.569	0.615	0.615
14	0.717	0.786	0.698	0.720	0.721	0.788	0.787
15	0.656	0.645	0.639	0.671	0.713	0.671	0.710
16	0.557	0.555	0.546	0.573	0.604	0.575	0.607
17	0.673	0.669	0.725	0.735	0.735	0.738	0.738
18	0.712	0.698	0.747	0.787	0.788	0.784	0.784
19	0.647	0.632	0.632	0.663	0.687	0.662	0.688
20	0.657	0.645	0.644	0.672	0.698	0.672	0.698

**Table 11:** Multi group confirmatory factor analysis based on PTSD models after removing item 8.

**Models**		**Goodness of fit indexes**					
	**χ^2^ (df)**	**CFI**	**TLI**	**RMSEA**	**RMSEA 90% CI**	**SRMR**	**BIC**
DSM-5	965.454 (146)^**^	0.936	0.925	0.061	[0.057, 0.065]	0.0394	1,287.645
Anhedonia and affect	850.662 (146)^**^	0.945	0.935	0.056	[0.053, 0.060]	0.0364	1,172.853
Dysphoria	1,013.845 (146)^**^	0.932	0.920	0.063	[0.059, 0.066]	0.0406	1,336.036
Dysphoric arousal	840.464 (142)^**^	0.945	0.934	0.057	[0.053, 0.061]	0.0373	1,191.945
Externalizing behaviors	817.863 (137)^**^	0.946	0.933	0.057	[0.054, 0.061]	0.0369	1,205.956
Anhedonia	653.471 (137)^**^	0.959	0.949	0.050	[0.046, 0.054]	0.0313	1,041.564
Hybrid	622.443 (131)^**^	0.961	0.950	0.050	[0.046, 0.054]	0.0306	1054.471

** *p = 0.000*.

**Table 12:** Standardized estimates for factor loadings after removing item 8.

**Items**	**DSM V**	**Anhedonia and affect**	**Dysphoria**	**Dysphoric arousal**	**Externalizing behaviors**	**Anhedonia**	**Hybrid**
1	0.710	0.709	0.709	0.710	0.710	0.709	0.709
2	0.722	0.722	0.722	0.723	0.723	0.723	0.723
3	0.714	0.714	0.714	0.714	0.714	0.714	0.714
4	0.723	0.723	0.722	0.722	0.722	0.723	0.723
5	0.766	0.766	0.765	0.765	0.765	0.765	0.765
6	0.911	0.911	0.912	0.911	0.911	0.908	0.908
7	0.884	0.883	0.883	0.883	0.883	0.887	0.886
9	0.585	0.570	0.574	0.587	0.587	0.606	0.603
10	0.574	0.574	0.565	0.572	0.572	0.605	0.605
11	0.639	0.643	0.639	0.633	0.634	0.661	0.662
12	0.719	0.774	0.693	0.719	0.718	0.768	0.769
13	0.567	0.609	0.557	0.573	0.573	0.613	0.613
14	0.730	0.787	0.705	0.733	0.733	0.790	0.789
15	0.658	0.650	0.643	0.674	0.719	0.675	0.717
16	0.554	0.549	0.542	0.570	0.600	0.570	0.601
17	0.674	0.668	0.727	0.734	0.734	0.738	0.738
18	0.713	0.697	0.749	0.788	0.788	0.784	0.784
19	0.646	0.628	0.628	0.661	0.686	0.660	0.688
20	0.657	0.645	0.642	0.673	0.699	0.673	0.698

## Discussion

Using epidemiology data about violence and mental health among Iraqi and Syrian displaced people, the current study examined the factor structure and cultural invariance of the DSM-V and six alternative models for PTSD. Although the DSM-V and all other alternative models yielded an acceptable fit to the data, the anhedonia model was superior to all other models tested here. This finding is in line with most of the previous studies among Western (Armour et al., 2015, 2016a; Bovin et al., 2016) and non-Western (Liu et al., 2014a,b; Yang et al., 2017; Specker et al., 2018) traumatized populations. It also converges with the conclusions of a recent systematic literature review (Armour et al., 2016b), as this review suggested separating criterion D into a negative affect factor and an anhedonia factor.

The current study is the first study that addresses the validity of the structure of PTSD based on the PCL-5 among diverse languages, nationalities, and ethnicities in the Middle East. Configural, metric, and scalar invariances were obtained by applying anhedonia and hybrid models. This result, along with results from CFAs in the full sample, provides further support for the empirical validity of the anhedonia and hybrid model. However, Armour et al. (2016b), identified the limitations of the anhedonia and hybrid models as they have more than one factor with two items only. Factors with few items can be problematic in terms of statistics, since at least three items per factor have been recommended before. According to Marsh et al. (1998), however, this limitation does not need to be applied in analyses that have large sample sizes. Thus, given that 1,514 displaced people participated in the current study, we can conclude that despite few items in some factors, D-factor models (anhedonia and hybrid) still provide superior fit when compared to DSM-V and other alternative models.

The present study used an exploratory factor analysis and introduced a new, empirically-supported model (the anhedonia and affect model) for the factor structure of PTSD. In this model, symptoms from B and C criterion were highly loaded in two separated factors, and symptoms in D and E factors were mixed across two different factors. The first part in this model is in harmony with the DSM-V, and all other proposed models, since intrusion and avoidance items load onto two specific factors. Regarding to criterion D and E, the anhedonia and affect model is partially in line with the recently-suggested models—the anhedonia (Liu et al., 2014a) and hybrid (Armour et al., 2015) models—that also gathered the same three symptoms in an anhedonia factor (diminished interest in activities, feelings of detachment from others, and inability to experience positive emotions). In this population and across populations worldwide it seems that there is unambiguous support for the three factors, intrusion, avoidance and anhedonia that should be considered in future revisions of the PTSD concept.

In the present study we investigated the correlation between PTSD symptoms clusters and symptoms of depression. We noted that the factors that hosted internalizing symptoms, such as dysphoric arousal, anhedonia, and negative affect, were all associated with depression symptoms higher than items and factors that described externalizing behaviors. This result indicates that a division of externalizing and internalizing symptoms, which had been proposed as a general factor structure of psychopathology, might be replicated within the PTSD category. However, this idea requires further elaboration since only limited externalizing symptoms had been assessed in this study. In particular there is no information about alcohol consumption (Claycomb Erwin et al., 2017) or aggression (Durham et al., 2016), which could be relevant for traumatized populations and would be required to test the relation of the factors to externalizing symptoms. This finding is also in line with previous studies (Armour et al., 2011, 2012, 2013; Ross et al., 2018) that also found a strong association between the dysphoric arousal factor and symptoms of depression. It is likely that a conceptual overlap and an item overlap both contribute to this correlation to some degree. Simms et al. (2002) indicated that symptoms, such as sleeping difficulties, irritability, and concentration difficulties are more likely to represent non-specific symptoms of general distress rather than hyperarousal, therefore they are quite similar to symptoms of depression and anxiety.

In line with most of the previous item level and factor analytic studies of PTSD (Wang et al., 2011, 2015b; Liu et al., 2014a,b; Vindbjerg et al., 2016), we observed that the psychogenic amnesia symptom (item 8) contributed poorly to all of the factors and to the overall structure of PTSD. In our study, the prevalence and standardized factor loading for psychogenic amnesia item was lower than all other PTSD symptoms. There is a long-standing debate about the validity of psychogenic amnesia as a core symptom of PTSD (for review, see Zoellner et al., 2013). The present study shows that by removing item 8 from CFAs the model fit for all models improved. Thus, the current analysis adds further doubts to the validity of this symptom for the PTSD diagnosis.

The study's instrument limits the results of the present study. Although a valid and reliable version of the PCL-5 was used in this study that was administered by trained local clinical psychologists and social workers, the PCL-5 is still a self-report and screening instrument with limited validity as a representative of DSM-V symptoms. Previous research showed that there is weak support for the DSM-V model when PCL-5 is applied (Krüger-Gottschalk et al., 2017), but further studies on the factor structure of PTSD using a clinical instrument, such as the Clinician-Administered PTSD Scale for the DSM-V (CAPS-5), are needed to provide significant information about the structure of PTSD's factors.

The current study is the first structural equation modeling study of PTSD factor structure among the displaced people in the Middle East. While the DSM-V and all other alternative models for PTSD reached an acceptable fit, the anhedonia model provided the best fit to the data, and the invariance of PTSD was obtained by applying the anhedonia and hybrid model. Overall, the current study provides further support for the global validity of the anhedonia and hybrid model of PTSD, and it contributes to filling the gap in knowledge about the validity of PTSD symptoms clusters among non-Western populations.

## Ethics Statement

The research and its procedures were approved by the Ethical Review Committees of Bielefeld University in Germany and Koya University in the Kurdistan Region of Iraq.

## Author Contributions

HI and FN conceptualized and designed the study. HI was the co-principal investigator and project manager, trained the local interviewers, supervised data acquisition, performed the data preparation and statistical analysis as well as the interpretation of data, and wrote the manuscript. AI was the co-principal investigator. CC contributed to the interpretation of data. FN was the chief investigator for this study, supervised data analyses, participated in the interpretation of the data, and critically revised the manuscript for important intellectual content.

### Conflict of Interest Statement

The authors declare that the research was conducted in the absence of any commercial or financial relationships that could be construed as a potential conflict of interest.
